# ABT-737 reverses the acquired radioresistance of breast cancer cells by targeting Bcl-2 and Bcl-xL

**DOI:** 10.1186/1756-9966-31-102

**Published:** 2012-12-23

**Authors:** Ji-Yu Li, Yu-Yang Li, Wei Jin, Qing Yang, Zhi-Ming Shao, Xing-Song Tian

**Affiliations:** 1 Department of Breast and Thyroid Surgery, Provincial Hospital Affiliated to Shandong University, 324 Jingwu-Weiqi Road, Jinan, 250021, People’s Republic of China; 2 Department of Breast Surgery, The Second Hospital of Shandong University, 247 Beiyuan Road, Jinan, 250033, People’s Republic of China; 3 Department of Breast Surgery, Breast Cancer Institute, Shanghai Cancer Center, Shanghai Medical College, Fudan University, 399 Ling-Ling Road, Shanghai, 200032, People’s Republic of China

**Keywords:** ABT-737, Breast cancer, Acquired radioresistance, Radiation, Bcl-2, Bcl-xL

## Abstract

**Background:**

Acquired radioresistance of cancer cells remains a fundamental barrier to attaining the maximal efficacy of radiotherapy for the treatment of breast cancer. Anti-apoptotic proteins, such as Bcl-2 and Bcl-xL, play an important role in the radioresistance of cancer cells. In the present study, we aimed to determine if ABT-737, a BH3-only mimic, could reverse the acquired radioresistance of the breast cancer cell line MDA-MB-231R by targeting Bcl-2 and Bcl-xL.

**Methods:**

The radiosensitivity of MDA-MB-231 and MDA-MB-231R cells was compared using colony formation assays. Reverse-transcription PCR and western blot were performed to detect the expression of Bcl-2 and Bcl-xL in the cancer cell lines. Annexin V flow cytometric analysis and caspase-3 colorimetric assay were used to evaluate apoptosis of the cancer cells. Cell viability was measured using the Cell Counting Kit-8. The animals used in this study were 4 to 6-week-old athymic female BALB/c nu/nu mice.

**Results:**

The MDA-MB-231R cells were more radioresistant than the MDA-MB-231 cells, and Bcl-2 and Bcl-xL were overexpressed in the MDA-MB-231R cells. While ABT-737 was able to restore the radiosensitivity of the MDA-MB-231R cells *in vitro* and *in vivo* experiment, it was not able to enhance the radiosensitivity of the MDA-MB-231 cells. In addition, ABT-737 increased radiation-induced apoptosis in the MDA-MB-231R cells. Bcl-2 and Bcl-xL were down regulated in the MDA-MB-231R cells following treatment with ABT-737.

**Conclusions:**

Targeting of the anti-apoptotic proteins Bcl-2 and Bcl-xL with ABT-737 may reverse the acquired radioresistance of MDA-MB-231R cells in vitro and in vivo. These findings suggest an attractive strategy for overcoming the acquired radioresistance of breast cancer cells.

## Background

Breast cancer is one of the most common malignant cancers among women worldwide. In 2012, an estimated 220,000 individuals were diagnosed with breast cancer and the mortality associated with breast cancer is nearly 40,000 in the United States [1]. Radiotherapy plays an important role in the treatment of breast cancer. Several randomized clinical trials have shown that improved disease-free and overall survival rates were improved by the addition of radiotherapy in the treatment of women with breast cancer [2-5]. However, tumor radioresistance remains a fundamental barrier to attaining maximal efficacy with radiotherapy for the treatment of breast cancer. Radioresistance may be present at the beginning of therapy, causing the patients to fail to respond to treatment (intrinsic radioresistance), or it may emerge over time during radiotherapy treatment (acquired radioresistance). Fractionated radiation (FR) is often used in radiotherapy to facilitate the recovery of normal tissues. Cancer cells may acquire radioresistance during fractionated radiotherapy, which results in treatment failure. Overcoming the acquired radioresistance of breast cancer could improve the outcome of breast cancer patients who receive radiotherapy.

Apoptosis, or programmed cell death, is the mechanism of radiation-induced cancer cell death [6,7]. It is regulated by complex interactions between anti-apoptotic proteins such as Bcl-2, Bcl-xL and Mcl-1, and pro-apoptotic proteins including Bax, Bak, Bad and Bim [8,9]. Down regulation of anti-apoptotic proteins can promote apoptosis and enhance the radiosensitivity of cancer cells [10-13]. The disruption of anti-apoptotic pathways is a novel target for overcoming radioresistance in breast cancer.

ABT-737 is a rationally designed small molecule that binds with high affinity to Bcl-2 and Bcl-xL and antagonizes their anti-apoptotic function, thereby inducing apoptosis in many cancer cell types [14,15]. Recently, an increasing number of studies have focused on the role of ABT-737 in cancer therapy.ABT-737 have been shown to reverse acquired paclitaxel resistance in breast cancer cell lines [16]. Combined with rapamycin, ABT-737 has been shown to enhance the radiosensitivity of non-small cell lung tumors by inducing apoptosis [16,17].

To our knowledge, there have been no prior studies investigating the effect of ABT-737 in combination with radiotherapy for the treatment of breast cancer. In the present study, we addressed whether ABT-737 could reverse the acquired radioresistance in breast cancer cells with the aim of develop a new strategy to address the serious clinical problem of acquired radioresistance in breast cancer.

## Methods

### Cell culture, materials and reagents

The human breast cancer cell line MDA-MB-231 was purchased from the American Type Culture Collection. The cells were grown in Leibovitz’s L-15 medium (11415–064, GIBCO) supplemented with 10% fetal bovine serum (FBS) (10099–158, GIBCO) and maintained in a humidified 5% CO_2_ atmosphere at 37°C. ABT-737 was purchased from Santa Cruz Biotechnology, Inc (SC-207242).

### Generation of radioresistant cells

MDA-MB-231 cells (1 × 10^6^) were plated in 75 cm^2^ culture flasks and irradiated with 4Gy of γ-rays using a Theratron Cobalt-60 treatment unit at a dose rate of 1 Gy per minute when the cells were at approximately 60% confluence in the culture flask. Immediately following irradiation, the culture medium was renewed, and the cells were returned to the incubator. When the MDA-MB-231 cells reached approximately 90% confluence, they were trypsinized, counted and passaged into new culture flasks. Again, the cells were treated with 4 Gy γ-rays when they reached approximately 60% confluence. The irradiation was performed 13 times for a total dose of 50 Gy (irradiated with 2 Gy of γ-rays at the final irradiation) over 5 months. The parental cells were trypsinized, counted and passaged under the same conditions without irradiation.

### Clonogenic assay for radiosensitivity

The cells were seeded in 6-well cell culture plates and incubated for 2 weeks at 37°C after the receiving various doses of irradiation. The colonies were fixed with pure ethanol and stained with 1% crystal violet, washed and air-dried. Colonies consisting of 50 or more cells were counted as clonogenic survivors. The surviving fraction (SF) was calculated by dividing the number of colonies by the number of cells seeded and then multiplying by the plating efficiency. Triplicate experiments were performed independently.

### Western blottings

Western blottings using rabbit anti-human Bcl-2 antibody (#2876, Cell Signalling Technology) and rabbit anti-human Bcl-xL antibody (556361, BD Biosciences) were performed according to standard protocols. Chemiluminescent detection was performed and images were captured by the FUJIFILM LAS-3000 system (Fujifilm, Tokyo, Japan).

### Extraction of RNA and RT –PCR

Total RNA was extracted using TRIzol reagent (Invitrogen) according to the manufacturers’ recommendations. RT-PCR(Reverse-Transcription PCR) was used to compare the relative mRNA expression of Bcl-2 and Bcl-xL in breast cancer cell lines. The primer sequences used were: Bcl-2, sense, 5^′^- GTGAACTGGGGGAGGATTGT-3^′^ and antisense, 5^′^- GGAGAAATCAAACAGAGGCC-3^′^ and Bcl-xL_,_ sense, 5^′^-CCCAGAAAGGATACAGCTGG-3^′^ and antisense, 5^′^- GCGATCCGACTCACCAATAC-3^′^. Thirty-two cycles of PCR were performed using the program of 30 s at 94°C, 30 s at 56°C and 1min at 72°C. The PCR products were electrophoresed on 2% agarose gel and imaged using a ChemiImag 5500 Imaging System (Alpha Innotech, San Leandro, CA, USA).

### Apoptosis assay

MDA-MB-231 and MDA-MB-231R cells (1 × 10^6^) were plated in 10 mm dishes for each data point. Following incubation overnight at 37°C, the cells were treated with ABT-737 (1 μM, 24 hours) and irradiated with 4 or 12 Gy. After 24 h, apoptotic analyses were performed by flow cytometry, as described previously [18], using a FACS Calibur system (Becton Dickinson Biosciences, San Diego, CA) with ModFit LT™ software (Verity Software House, Inc., Topsham, ME). The apoptotic cells were analyzed by using quadrant statistics on the propidium iodide-negative and Annexin V-positive cells.

### Caspase-3 colorimetric assay

The cells were collected and washed with phosphate-buffer saline (PBS, pH 7.2). After centrifugation, the caspase 3 colorimetric assays were performed according to the manufacturer’s specifications (ab39401, Abcam) using a Sunrise Microplate Reader(Tecan US, Inc.,Charlotte, NC).

### Cell viability

Cell viability was evaluated using Cell Counting Kit-8 (CCK-8; Dojindo Molecular Technologies Inc., Gaithersburg, MD) assay. The cells were plated in 96-well plates at 1 × 10^4^ cells/well with media only, media with ABT-737 (1 μM) or DMSO, which were changed with media 24 hours later. To evaluate cell viability, 10 μl of CCK-8 was added per well, and the cells were incubated for an additional 4 hours, Following the incubation, the absorbance at 450 nm was recorded using a 96-well plate reader (Sunrise Microplate Reader, Tecan US, Inc.,Charlotte, NC).

### Animal experiments

The animals used in this study were 4 to 6-week-old athymic female BALB/c nu/nu mice which were provided by the Shanghai Institute of Materia Medica, Chinese Academy of Science. MDA-MB-231R cells (10^6^) were implanted into the mammary fat pad. Twenty-four nude mice were divided into 4 groups for this study, with each group having 6 mice. The four groups were the ABT-737 group, the ABT-737 plus radiation group, the DMSO plus radiation, and the DMSO group. Fourteen days following tumor inoculation, DMSO and ABT-737 were administered intraperitoneally at doses of 20 mg/kg for 7 consecutive days. The mice receiving radiation were irradiated 1 hour after ABT-737 or DMSO treatment with 2 Gy daily over 5 consecutive days. The tumors on the mice were irradiated using γ-rays (Theratron 1000E Cobalt-60 treatment unit, Canada). The non-tumor parts of the mice were shielded with lead blocks. The rate of tumor growth was determined by plotting the means of two orthogonal diameters of the tumors, which were measured at 7-day intervals. The animals were monitored for tumor growth and general health every 2 days for up to 6 weeks. The tumor volumes were calculated using the following formula: volume = 0.52 × width^2^ × length. The animals were sacrificed and autopsied 6 weeks after tumor inoculation. All studies on mice were conducted in accordance with the National Institutes of Health ‘Guide for the Care and Use of Laboratory Animals’. The study protocol was approved by Shanghai Medical Experimental Animal Care Committee.

### Statistical analysis

Statistical analysis was performed using the Statistical Package for the Social Sciences (SPSS) software Version 11.5 for Windows (SPSS Inc., Chicago, IL). ANOVA and Student’s t-tests were conducted to determine the statistical significance of the differences between the experimental groups. A value of *p* < 0.05 was considered statistically significant. The graphs were created using GraphPad Prism 5.

## Results

### Morphology and radiosensitivity of MDA-MB-231R cells

**Figure 1:**
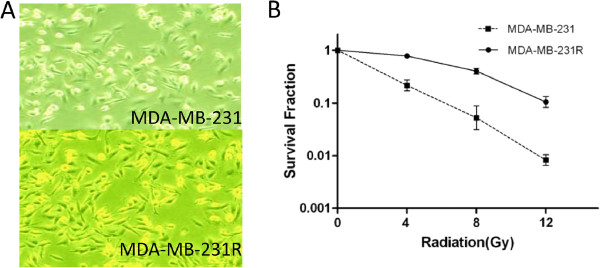
**Morphology and radiosensitivity of MDA-MB-231R cells. **(**A**) No obvious change in the cell morphology was observed following radiation. (**B**) The radioresistant MDA-MB-231R cells had a higher survival rate than the non-radioresistant MDA-MB-231 cells.

The radioresistant cells, designated MDA-MB-231R, were obtained by subjecting MDA-MB-231 cells to 5 months of fractioned irradiation with a total dose of 50 Gy and 10 additional passages without irradiation. No obvious change in the cell morphology was observed following irradiation (Figure 1A). The radiosensitivity of MDA-MB-231 and MDA-MB-231R cells were compared using a colony formation assay (Figure 1B). Each point on the survival curve represents the mean surviving fraction from triplicate experiments. As expected, the MDA-MB-231R cells had a higher survival rate than MDA-MB-231 cells, indicating that the MDA-MB-231R cells were more radioresistant than the MDA-MB-231 cells.

### Bcl-2 and Bcl-xL are overexpressed in MDA-MB-231R cells

**Figure 2:**
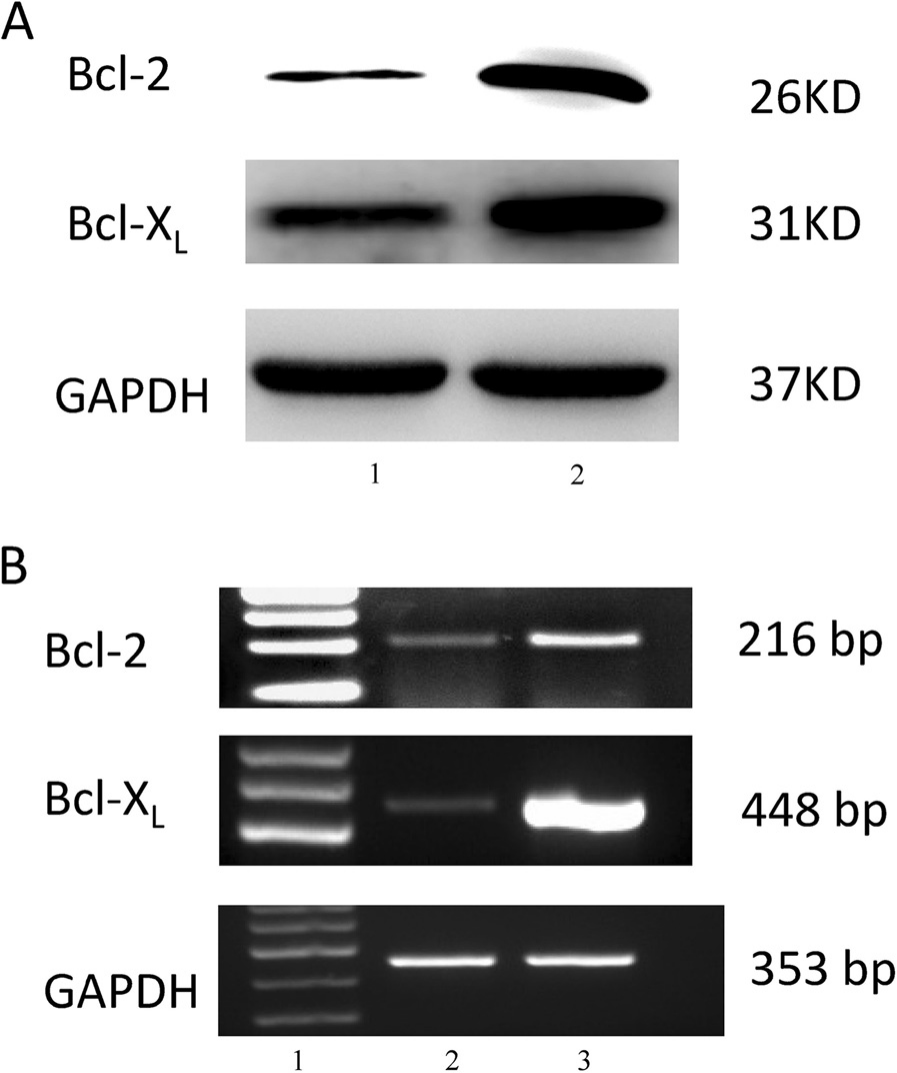
**Bcl-2 and Bcl-xL are overexpressed in MDA-MB-231R cells. **(**A**) Western blot analysis showed that the anti-apoptotic proteins Bcl-2 and Bcl-xL are overexpressed in the MDA-MB-231R cells compared with MDA-MB-231 cells. Lane 1, MDA-MB-231 cells; lane 2, MDA-MB-231R cells. (**B**) RT-PCR analysis further confirmed an overexpression of Bcl-2 and Bcl-xL in the MDA-MB-231R cells. Lane 1, marker; lane 2, MDA-MB-231cells; lane 3, MDA-MB-231R cells.

Because anti-apoptotic proteins could enable the radio resistance of the cancer cells, we investigated whether the expression of Bcl-2 and Bcl-xL, important proteins involved in apoptosis, were altered in the MDA-MB-231R cells. Western blotting showed a significant increase in Bcl-2 and Bcl-xL expression in the MDA-MB-231R cells compared to MDA-MB-231 cells (Figure 2A). These results were further verified by RT-PCR (Figure 2B). These findings suggest that the overexpression of anti-apoptotic proteins, including Bcl-2 and Bcl-xL, is important for the acquisition of radioresistance by cancer cells.

### ABT-737 restores the radiosensitivity of MDA-MB-231R cells

**Figure 3:**
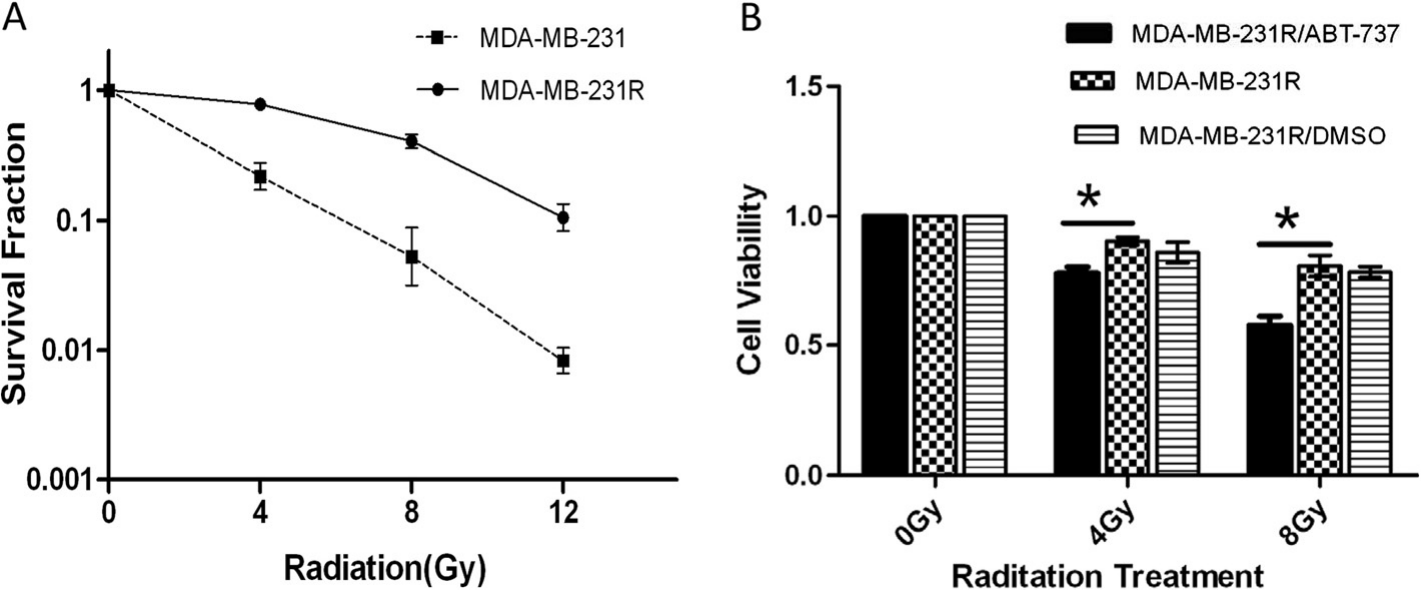
**ABT-737 restores the radiosensitivity of MDA-MB-231R cells. **(**A**) The colony forming ability of the MDA-MB-231R cells was significantly decreased following 1 μM ABT-737 for 24 hours. (**B**) Cell viability assays demonstrated that pre-treatment with ABT-737 increases radiation-induced cell death in the MDA-MB-231R cells. *P < 0.05. Columns, mean of three independent experiments; bars, SD.

Colony formation assays were used to determine if ABT-737 could restore the radiosensitivity of the MDA-MB-231R cells. As shown in Figure 3A, the colony-forming ability of the MDA-MB-231R cells greatly decreased following treatment with 1 μM of ABT-737 for 24 hours. This result indicated that the radiosensitivity of the MDA-MB-231R cells was significantly increased following treatment with ABT-737. The cell viability assays demonstrated that ABT-737 was able to reverse the acquired radioresistance of the MDA-MB-231R cells. The radiation-induced decrease in cell viability was enhanced by a 24 hour pre-treatment with 1 μM ABT-737 (Figure 3B).

### ABT-737 does not enhance the radiosensitivity of MDA-MB-231 cells

**Figure 4:**
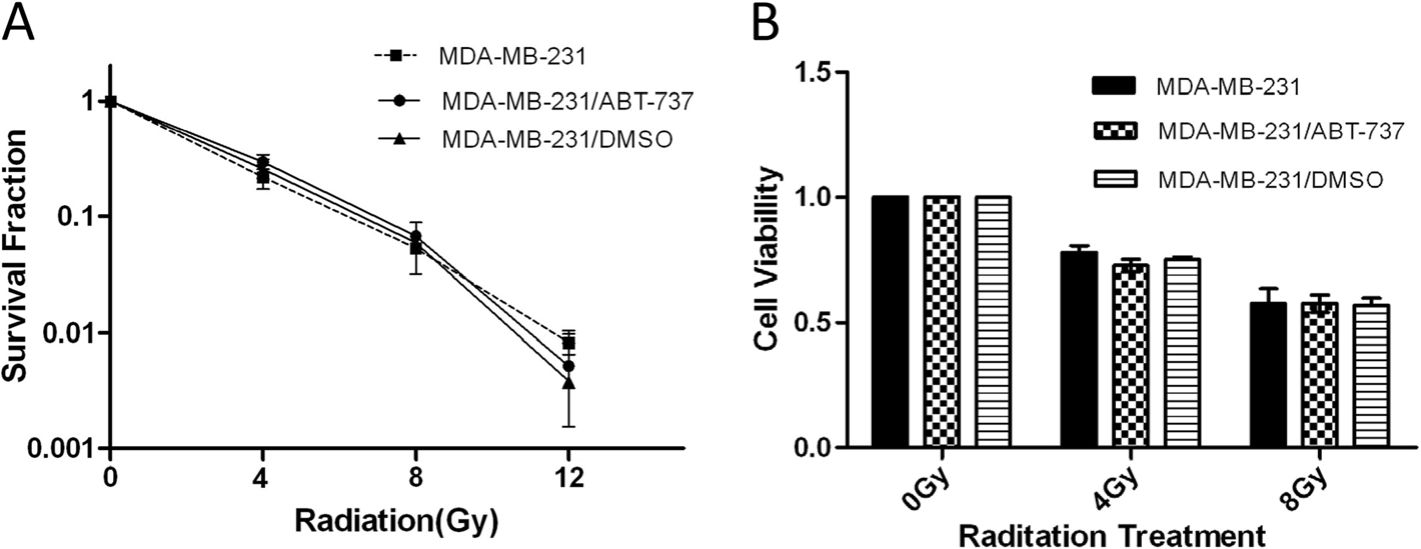
**ABT-737 does not enhance the radiosensitivity of MDA-MB-231 cells. **(**A**) Survival curves of the MDA-MB-231 cells with or without ABT-737 treatment following radiation. (**B**) Cell viability assays demonstrated that pre-treatment with ABT-737 does not increase radiation-induced cell death in the MDA-MB-231 cells. Columns, mean of three independent experiments; bars, SD.

We further investigated whether ABT-737 could enhance the radiosensitivity of MDA-MB-231 cells. The colony formation assays revealed that the radiosensitivity of the MDA-MB-231 cells did not change significantly following treatment with ABT-737 (Figure 4A). The cell viability assays further demonstrated that ABT-737 did not enhance the radiosensitivity of MDA-MB-231 cells (Figure 4B).

### ABT-737 increases the radiation-induced apoptosis of MDA-MB-231R cells

**Figure 5:**
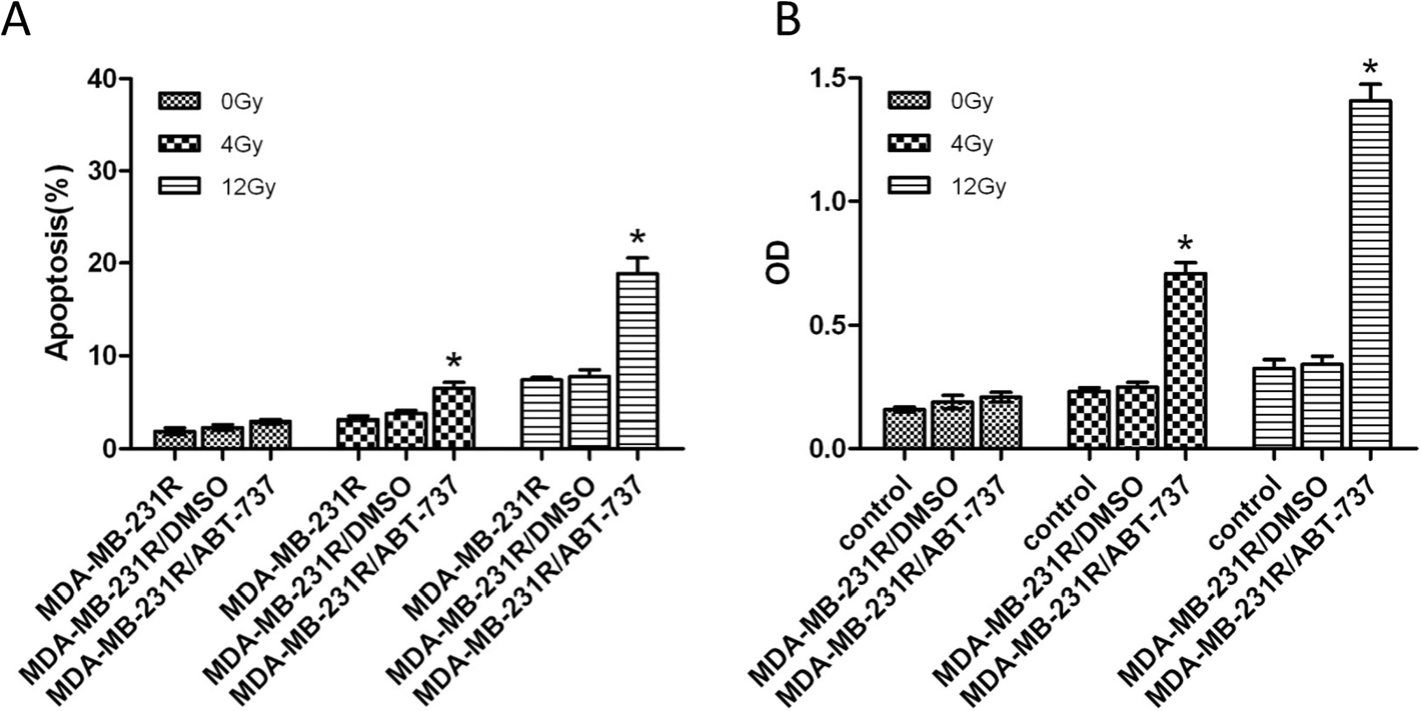
**ABT-737 increases the radiation-induced apoptosis of MDA-MB-231R cells. **(**A**) The proportion of Annexin V-positive and propidium iodide-negative cells (apoptotic cells) was significantly higher in the ABT-737-treated group compared to the untreated and DMSO control groups. (**B**) A caspase-3 colorimetric assay was performed to confirm our findings. The activity of caspase 3 was significantly upregulated after treatment with ABT-737. Columns, mean of three independent experiments; bars, SD.

Annexin V flow cytometric analysis was used to determine if ABT-737 could enhance the radiation-induced apoptosis of MDA-MB-231R cells. The MDA-MB-231R cells were treated with DMSO or 1 μM ABT-737 for 24 hours prior to radiation (0, 4, or 12 Gy). As shown in Figure 5A, the proportion of Annexin V-positive and propidium iodide-negative cells (apoptotic cells) was significantly higher in the ABT-737-treated group than in the untreated and DMSO control groups. A caspase-3 colorimetric assay was performed to confirm our findings. The activity of caspase 3 was significantly upregulated after treatment with ABT-737.These data suggest that ABT-737 increased the radiation-induced apoptosis of the MDA-MB-231R cells.

Bcl-2 and Bcl-xL are down-regulated in MDA-MB-231R cells and are unchanged in MDA-MB-231 cells following ABT-737 treatment.

**Figure 6:**
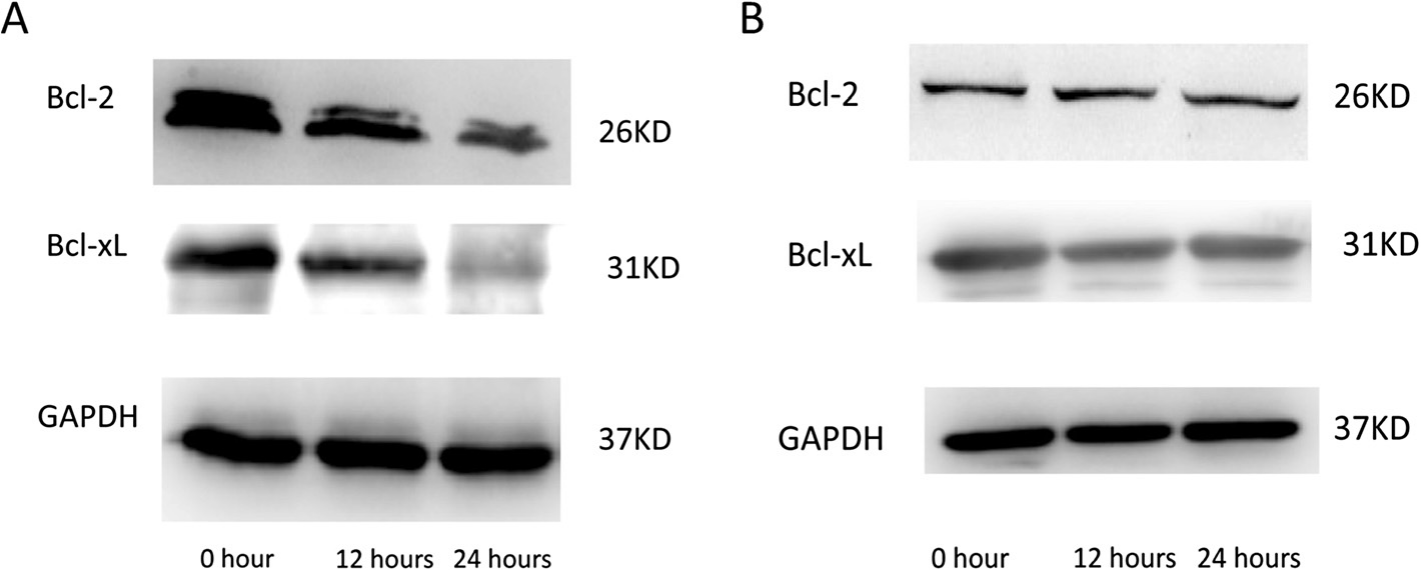
**Bcl-2 and Bcl-xL are down-regulated in MDA-MB-231R cells and are unchanged in MDA-MB-231 cells following ABT-737 treatment. **(**A**) The expression of Bcl-2 and Bcl-xL in MDA-MB-231R cells gradually decreased over 24 hours when treated with 1 μM of ABT-737. (**B**) In contrast, the expression of Bcl-2 and Bcl-xL in the MDA-MB-231 cells did not change after ABT-737 treatment.

To evaluate the effect of ABT-737 on the apoptotic pathway, we examined the expression of Bcl-2 and Bcl-xL in MDA-MB-231R and MDA-MB-231 cells following treatment with ABT-737. We found that ABT-737 directly downregulated Bcl-2 and Bcl-xL expression in the MDA-MB-231R cells in a time-dependent manner. The expression of Bcl-2 and Bcl-xL in the MDA-MB-231R cells gradually decreased over 24 hours of treatment with 1 μM ABT-737 (Figure 6A). In contrast, the expression of Bcl-2 and Bcl-xL in the MDA-MB-231 cells did not change after ABT-737 treatment (Figure 6B). These results indicate that ABT-737 reversed the acquired radioresistance of the MDA-MB-231R cells by downregulating the expression of Bcl-2 and Bcl-xL.

### ABT-737 can reverse the acquired radioresistance of breast cancer cells *in vivo*

**Figure 7:**
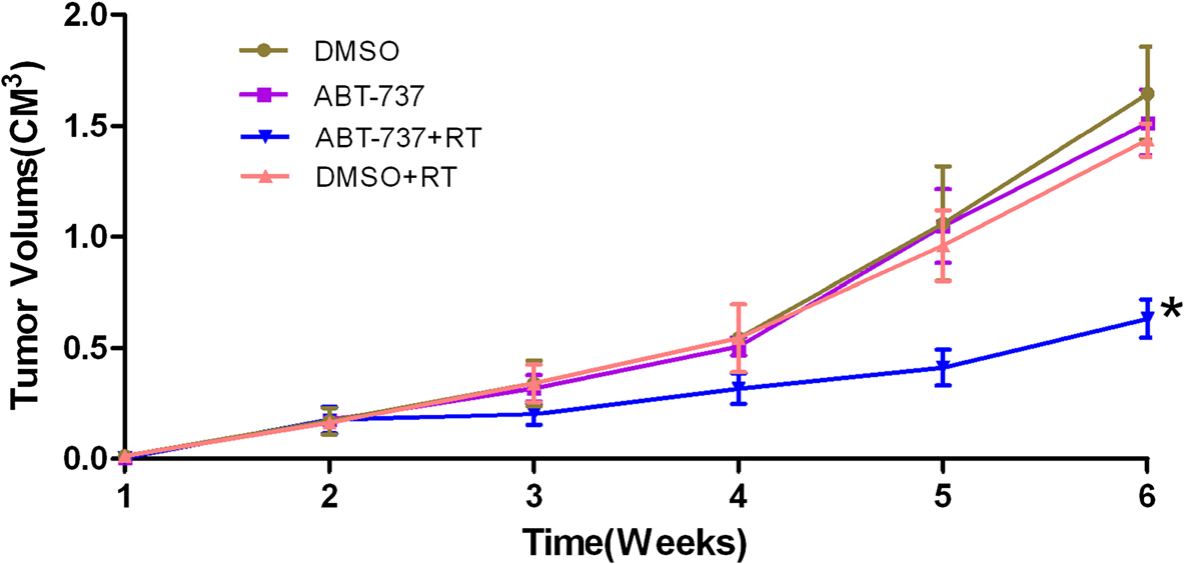
**ABT-737 can reverse acquired radioresistance of breast cancer cells in vivo. **The method of xenograft tumor volume measurement, radiation and reagent administration are described in Methods section. The tumor volumes in the ABT-737 plus radiation group were significant smaller than those in the DMSO plus radiation group (*P <* 0.01). However, the growth curves of the tumors in the DMSO group, the DMSO plus radiation group and the ABT-737 group were very similar. In this study, six mice per group were used. Points, mean volumes; bars, SD.

To investigate whether ABT-737 could reverse acquired radioresistance of breast cancer cells *in vivo*, we used an orthotropic xenograft tumor model in nude mice. As shown in Figure 7, the MDA-MB-231R tumors in the DMSO group of mice were similar to the tumors in the DMSO plus radiation group. This indicated that the MDA-MB-231R tumors were radioresistant. The tumors in the ABT-737 group were not significantly different from those in the DMSO group. The tumors in the ABT-737 plus radiation group grew at a slower rate than the tumors in the DMSO plus radiation group. Taken together, these results suggested that ABT-737 could reverse the radioresistance of MDA-MB-231R tumors.

## Discussion

Fractionated radiation (FR) is used often in radiotherapy treatment to facilitate the recovery of normal tissues, while the repair of cancer cells is generally less efficient between fractions. However, the acquired radioresistance of cancer cells is thought to occur during the repopulation of the tumor during the long-term FR [19]. The proliferating cancer cells that repopulate the tumor may be different subpopulation with a different genotype that confers radioresistance. Cancer cells with acquired radioresistance many survive during radiotherapy and lead to additional cancer recurrence after radiation therapy, thus limiting the effectiveness of radiation therapy. Therefore, to promote better outcomes for patients undergoing radiotherapy, an effective strategy may be to target the cells acquired radioresistance.

In the present study, the MDA-MB-231R cells were obtained after fractionated radiation with total dose of 50 Gy and were cultured without radiation for the next 10 passages. The radioresistance of MDA-MB-231R cell line was determined using a colony-forming assay. The results of Western blot analysis showed that the anti-apoptotic proteins Bcl-2 and Bcl-xL were overexpressed in the MDA-MB-231R cells that had acquired radioresistance. RT-PCR analysis confirmed that the expression of the anti-apototic genes Bcl-2 and Bcl-xL were upregulated in the radioresistant MDA-MB-231R cells and overexpressed compared with their parental cell line. The overexpression of anti-apoptotic proteins in the Bcl-2 family is frequently observed in many different tumor types and has been associated with resistance to radiotherapy [20,21]. However, the molecular mechanism underlying the acquired radioresistance of cancer cells remains unclear. Several mechanisms are thought to contribute to the acquired radioresistance, including mutated p53 [22], amplification of DNA repair genes [23], overexpression of the cell-cycle regulator protein, cyclin D1 [19] and activation of pro-survival oncogenes such as EGFR [24]. The overexpression of Bcl-2 and Bcl-xL in the MDA-MB-231R cells indicated that these anti-apoptotic proteins play an important role in the acquisition of radioresistance.

The expression of anti-apoptotic proteins is closely related to the radiosensitivity of cancer cells, and targeting these proteins could be an effective method to overcome radioresistance. An et al. overcame the radioresistance of prostate cancer cells by using HA14-1, a novel Bcl-2 inhibitor [10]. Anai et al. demonstrated that down regulation of Bcl-2 could induce radiation sensitivity in prostate cancer cells [11]. The expression levels of the anti-apoptotic proteins are also correlated with the outcome of patients who received radiotherapy. Yang et al. [25] reported that Bcl-2 expression is associated with an increased risk of the local recurrence in patients with early breast cancer that received breast conservative surgery and radiotherapy. AT-101, a small molecule inhibitor of the Bcl-2 family members, enhanced the radiation-induced apoptosis of human leukemia cells [26]. We proposed that targeting the overexpression of Bcl-2 and Bcl-xL may be an effective way to overcome the acquired radioresistance of cancer cells. In this study, it was observed that following treatment with 1 μM ABT-737 for 24 hours, the colony formation ability of MDA-MB-231R cells decreased greatly and the radiation-induced apoptosis increased. These data suggested that ABT-737 could reverse the acquired radioresistance of MDA-MB-231R cells by increasing radiation-induced apoptosis. *In vivo*, the growth tumors in the ABT-737 plus radiation group were reduced compared with the DMSO plus radiation group. However, in contrast to the results obtained with the MDA-MB-231R cells, ABT-737 did not enhance the radiosensitivity of the MDA-MB-231 cells. This could be attributed to the down regulation of Bcl-2 and Bcl-xL expression observed in MDA-MB-231R cells, but not in MDA-MB-231 cells following ABT-737 treatment (Figure 6A and B). The expression levels of Bcl-xL and Bcl-2 in the MDA-MB-231 cells were very low, and treating them with ABT-737 did not down regulate their expression. Although treatment with ABT-737 did not enhance the radiosensitivity of the MDA-MD-231 cells, it reversed the acquired radioresistance of the MDA-MD-231R cells, making them more likely to be killed by radiation treatment. Eliminating these radioresistant cancer cells is perhaps the most effective method for decreasing the recurrence of cancer following radiotherapy.

This is the first study to show that ABT-737 down regulated the expression of Bcl-2 and Bcl-xL in cancer cells in a time-dependent manner. ABT-737, a rationally designed small molecule binds with high affinity to Bcl-2 and Bcl-xL, thereby antagonizing their anti-apoptotic functions and inducing apoptosis in many types of cancer cell. ABT-737 binds to the multi-domain, anti-apoptotic Bcl-2 family member proteins to prevent them from sequestering the pro-apoptotic BH3-only proteins. In the present study, we found that ABT-737 down regulated the expression of Bcl-2 and Bcl-xL in MDA-MB-231R cells in a time-dependent manner. Similar results were obtained using SK-BR-3 and MCF-7 cells (data not shown). The down regulation of those anti-apoptotic proteins by ABT-737 may at least partly explain its ability to reverse the acquired radioresistance of the MDA-MB-231R cells. Further studies are required to determine the mechanisms underlying this phenomenon.

In summary, treatment with ABT-737 reversed the acquired radioresistance of the MDA-MB-231R cells both *in vitro* and *in vivo*. The data indicate the potential benefit of ABT-737 treatment in conjunction with radiotherapy for breast cancer treatment and suggest a new strategy for improving the effectiveness of radiotherapy.

## Conclusions

In summary, our results suggest that targeting the anti-apoptotic proteins Bcl-2 and Bcl-xL with ABT-737 may reverse the acquired radioresistance of MDA-MB-231R cells *in vitro* and *in vivo*. These findings suggest an attractive strategy for overcoming the acquired radioresistance of breast cancer.

## Competing interests

The authors declared that they have no conflict of interest.

## Authors' contributions

XST and ZMS designed research; JYL, WJ, YYL and QY performed research; JYL, YYL analyzed data; JYL and WJ wrote the paper. All authors read and approved the final manuscript.
